# The Artistic Spirit in Medicine

**Published:** 1897-01

**Authors:** Stuart Close

**Affiliations:** Brooklyn, N. Y.


### THE ARTISTIC SPIRIT IN MEDICINE.

Stuart Close, M. D., Brooklyn, N. Y.

(Read before the Brooklyn Hahnemannian Union.)

In Homœopathy, as in other branches of art, science, or religion, many obstacles beset the path of one who sets before himself a high ideal and strives to attain it.

The natural inertia of the flesh, the desire for ease, the aversion to hard work, the ease with which routine habits are formed, the lack of appreciation of our best efforts, the temptations of wealth and pleasure which flatter and promise so much if we will only yield certain vital points, all demand unceasing vigilance and struggle as the price of victory.

The impatience, the short-sightedness, and the ingratitude of the ordinary patient who is so often longing in his heart for the palliative "flesh-pots of Egypt," and demanding the forbidden meat at our hands, is disheartening to one who is conscientiously trying to promulgate truth, and apply Hahnemannian principles in the treatment of the sick.

To have one's most brilliant cures unrecognized and lightly dismissed with the remark, "Oh, he wasn't very sick that time!" while the blundering idiot who has been calling three times a day on a patient next door for the last three weeks in a case that ought to have been cured with one prescription, is lauded as a wonderful doctor because he "pulled the patient through such a long and severe illness," is not calculated to arouse a high degree of enthusiasm, especially as the other fellow collects a smiling hundred dollars to our wry-faced ten after it is all over.

It is true there is also a *per contra;* that another class of patients are grateful, appreciative and kind, pay their bills promptly and cheerfully, and regard the doctor as their best friend. Making all due allowance, however, there is still a margin of need left, not only for "counting our blessings," but for diligently cultivating any elements or method by which a higher interest is awakened in our minds, and enthusiasm generated sufficient, at least, to keep us in the straight and narrow way of law and duty.

To use one's profession merely to gain wealth, fame, social or political position is ignoble, and inevitably results in destroying all interest in the higher phases of the profession, as well as technical facility therein. In art and science, as in religion, we must "seek first the Kingdom of God and His righteousness." This becomes not only practical, but easy, when we remember that "the Kingdom of God is *within*," and that "seeking it" is simply listening for and being guided by the behests of that "still small voice" within, which is ever prompting us to give embodiment and expression to the highest, the purest, the truest, and the best there is in us, in whatever sphere we may be. In doing this we are simply fulfilling the highest law of our being, and the reward is certain. When we are doing this with all our hearts, in our profession, as well as in our daily life, we may expect, and confidently rest in the assurance that the "all things" needful to our well-being shall be "added unto" us.

It may be well for us, therefore, to try to point out, suggestively, some of the higher phases and relations of our art, in order that they may inspire us with new interest and enthusiasm. Over against the discouraging things which force themselves upon our attention we will set some of the privileges and pleasures, and try to point out how the practice of our art may be made the means of our highest development.

The highest delight possible to man arises from the conscious exercise, in their legitimate spheres, of the powers and faculties with which he is endowed, for the purposes of his own highest development and the uplifting of his fellows.

There is no pleasure to be compared to that experienced by the true artist who has mastered the fundamental principles of his art, as he habitually makes practical application of those principles in the spirit of this high ideal, and witnesses the results.

What more legitimate or natural, therefore, than to recognize and cultivate the true artistic spirit in our work—the spirit which follows "art for art's sake," and which finds its highest and keenest delight in exercising the skill already acquired in constantly striving, by ever-improving methods, to attain its highest ideal?

There is something admirable even in disease. Nature is orderly, even in her disorders. A symptom-picture of disease does not spring full-formed into existence. It is a thing of orderly growth and development. Nature wields a delicate brush and pencil. Like a great artist, she first sketches in outlines, lightly, suggestively, in lines meaningless to the vulgar, but eloquent of what is to be to the trained eye of the artist.

Here she places a touch of color, there a stroke to indicate the perspective. Slowly or rapidly, according to circumstances, the creation grows until it stands complete, a harmonious whole, giving intelligible expression to an idea.

In this fact lies one source of satisfaction and enjoyment in the homœopathician's life. The pleasure derived from watching and studying the development of a symptom-picture, in the expectation of finding the curative, is closely akin to the enjoyment of an artist watching the growth of a picture on the easel under the master's hand, or in studying the finished canvas as he analyzes its features or copies it.

The homœopathic artist sits before Nature's easel—the human organism—watching a symptom-picture grow little by little until it takes on form and coherency, and assumes characteristic features; or, he is before the finished picture, an already developed case, perhaps a chronic case, and it becomes his duty to go back into the history of the case and analyze it, tracing its development step by step to the present. This he does by a process of examination which must be conducted according to the rules of evidence, and which requires, for its successful accomplishment, the exercise of profound knowledge of the laws of the human mind.

A true natural symptom-picture is a unity in which there is consistency, coherency, and character. The homœopathic artist is always guided by this fact in making his examinations. He searches until he finds this unity. If possible, he makes no prescription until he has not only found the complete image, but also its perfect correspondent in a remedy.

He can not only afford to wait and watch in patience, knowing this to be the shortest road to success, but he can do so gladly and confidently in the conscious possession of power to heal when all the facts have appeared and he has found the corresponding remedy according to the law of similars. Patience is only possible where confidence exists. Confidence is the result of faithful work, intelligently continued until mastery of the principles involved has been attained.

No one but a Hahnemannian ever sees a true, natural symptom-picture, and he only in his old and trusted families, who have learned wisdom and do not resort to the domestic case for every trifle. Other cases are so confused and changed by the action of inappropriate remedies that they have lost largely their natural characteristics, and have become a composite picture, blurred and vague, into which the natural and artificial symptoms enter in varying proportions.

It is in the examination of a patient by a Hahnemannian physician, as of a witness by a lawyer, that the most important, the keenest, the subtlest, and finest work is done. It is this that requires the most skill, the most delicacy and tact, and the most patience. For this reason it is the most delightful. Upon this all the rest depends. With the results of a partial or unskillful examination a master of the art of prescribing can do but little better than a tyro. Of two equally fine prescribers, the best examiner will be most successful in practice. Failures are often attributed to mistaken prescribing which are really due to poor or unsuccessful examining, but an unsuccessful examination is not always due to a fault in the examiner. The fault may be in the patient.

I recollect a case which had passed without benefit successively through the hands of Dr. Hering, Dr. Lippe, and Dr. David Wilson, of London, to finally come under Dr. Wells' care and be cured by him, not, as he explained to me, because he was able to prescribe any better than these celebrated men, or was a more skillful examiner, but simply because he was more fortunate in finding some symptoms they had failed to get. Something in this patient's relation with him, some question, some suggestion, some touch of sympathy so subtle as to be almost indefinable, brought out from the patient's subconscious memory facts long forgotten or not consciously observed until in Dr. Wells' presence, the possession of which enabled Dr. Wells to find the remedy and effect a cure.

To make a homœopathic examination successfully requires a knowledge of the laws by which the human mind and memory operate. The examiner must be able to unlock the halls of the patient's memory, by question and suggestion, until there shall be brought up above the threshold of consciousness those facts and experiences of the past which have been recorded upon the tablets of the subconscious memory, but which have been overlooked or forgotten through inattention or lapse of time. The ability to do this, to draw out from a patient such facts and experiences, to bring out those peculiar symptoms—"trifles light as air," and as elusive—upon which a successful prescription so often depends, stamps a man as an artist even more emphatically than the ability to select the curative after this has been done. Such ability is in some measure a natural gift, but even then it can best be perfected by a systematic study of psychology.

The physician must learn that no experience in life is ever lost; that no impression made upon the soul, through the medium of any of the senses, is ever effaced. All is indelibly recorded upon the subconscious memory. The power of recollecting it at will may be lost, but some time, unexpectedly, perhaps many years afterward, it comes back. During the delirium of fever, in dreams, while undergoing the experience of drowning, in the hypnotic condition, experiences long past the power of ordinary recollection to recall are once more firmly grasped.

Sound its keynote and the whole structure of the great Brooklyn bridge will vibrate in unison with it. Recall, by suggestion, to the patient's mind the keynote of fact or circumstance, and by the operation of the law of association of similar ideas you open up a train of symptoms or facts in his experience which will give you the basis for a prescription and the mastery of the case. Fail to do this, and you are left in the dark. The patient gets little help from you.

"The noblest study of man is man." Study of the laws of the human mind, so far as known, investigation of psychic phenomena, observing and recording all facts bearing upon the question of how to be able, at will, to come into such *rapport* with patients, in obscure cases, as to draw out these hidden symptoms is a most delightful and fascinating occupation. The field of psychology has just been fairly entered upon. Old ideas and theories which have cramped and limited the study of psychology are being rudely shaken and shattered by recent investigations. The new psychology, which is a spiritual, a dynamic psychology, as distinguished from the older materialistic system, is coming to the front. Unprejudiced study of the phenomena of hypnotism, telepathy, spiritism, mental suggestion, "mind cure," "christian science," "faith cure," hysteria, and psychic phenomena in general by men of high attainments and true scientific spirit, has done more to advance the science of psychology in the last ten years than in a century before.

All this is fertile and productive ground for the Hahnemannian, for it familiarizes him with the phenomena and laws of the human mind, and so enables him to make a better examination of his patients. As a field of study it is so interesting, so fascinating, that he may find relaxation and keenest pleasure while he is making solid scientific attainments. In it, oftener than in any other, is the old adage verified that "truth is stranger than fiction." It is the subject of the day and age, and in working upon it he is brought into relation, personal or otherwise, with the brightest and most progressive minds of the day while he is qualifying himself for the highest duties of his own profession.

If exercise makes a strong man, if fighting makes a good soldier, if struggles against temptation toughen the moral fibre, then may the Hahnemannian build himself up in the faith by combatting the errors and evils of the day in medical practice.

There is need enough for lifting up a high ideal before our own school. Too many of its members are in darkness. There is too much ignorance of its philosophy, too much carelessness, too much routine prescribing, too much running after old school fads and fancies, too little HOMŒOPATHY. There is too little recognition of the grand possibilities in straight Hahnemannian Homœopathy, of what is actually being done in curing the "incurably sick," and in saving many from mutilation by the surgeon's knife. It is too often taken for granted that a sickness must "run its course." Too often the physician is content to supinely accept the false and pernicious doctrine underlying the phrase "self-limited disease," and with superficial examination and prescriptions pilot the patient through long and weary weeks of illness, which might be cut short in the beginning if he would make himself master of the art of prescribing and apply himself to the case.

To their shame be it said, there are those in our own ranks, as well as in the old school, who deny the possibility of cutting short or *curing* a "self-limited disease," and whose only answer to the logic of facts is to deny the diagnosis, the *non credo* of small minds. It is a disgrace to a profession which calls itself "learned," and which professes to heal the sick, that such a term as "self-limited disease" is to be found in its vocabulary. It is a confession of ignorance and incompetency; ignorance of the law of cure, and incompetency to find it. This being so it becomes our duty, and privilege as well, to persistently proclaim the truth and to demonstrate in actual practice that there is something more than is commonly supposed in the first paragraph of Hahnemann's *Organon:* "The physician's highest and only calling is to *restore health to the sick, which is called Healing.*" This means something more than merely to nurse and coddle a patient through a long illness and collect a large bill at the end of it. It means that it is his duty and privilege, being possessed of the law of cure, to cut short and obliterate in its early stages an acute disease, even though it may be catalogued as "self-limited" in the text-books. This has been, is being, and can be done, in numerous cases from whooping cough to typhoid fever.

It is a significant, if pitiable, fact that a leading light in the medical world has lately declared, "we do not pretend to *cure*, we only *treat* the sick." It is also significant that the clearest sighted, the most progressive, and most honest minds of the old school of medicine are leaving the domain of therapeutics and entering surgery, hygiene, and sanitary science. Acknowledging their failure to cure, they say "we will improve conditions, we will prevent." Preventive medicine vies with "serum therapy" and the "microbe theory" in its claims to being the question of the day. As between these elements it is most devoutly to be hoped that preventive medicine, in so far as it depends upon hygiene and sanitary science, may win. It is at least neutral, clean, and wholesome.

The abominations of "serum therapy" are a stench in the nostrils of an outraged community. It is the era and apotheosis of filth. Syphilis, or "great pox," and its analogues, small-pox, cow-pox, horse-pox, and chicken-pox, scrofula, tuberculosis, cancer, hydrophobia, leprosy, cholera, diphtheria, scarlet fever, and measles are all being, or will be made to yield the essence of their filth in order that our health (?) boards may be able to control and treat us if we are sick, or render us "immune" if well. Already endowed with most extraordinary and tyrannical powers, they are constantly reaching out for more.* We are already largely at the mercy of a set of mercenary, unscrupulous, politico-medical demagogues, who have not hesitated in many instances to violate by force of arms, if necessary, the privacy of our homes or the sacredness of our persons in carrying out their nefarious schemes.

Against all this, as Hahnemannians, we set our faces like a flint. To this evil, selfish, materialistic philosophy, as applied in medicine, we oppose the pure, spiritual, dynamic philosophy of Hahnemann! It is like leaving a dismal tangled swamp, reeking with poisonous vapors and filled with loathsome reptiles, and coming out into the pure air, bright sunshine, and refreshing breezes of the fertile uplands to get upon the Hahnemannian plane in medicine. Here is no uncertainty, no wavering, no vague and misty speculation, but pure inductive reasoning from accurately observed facts, strict individualization, clear and positive statements, lofty ideals based upon right conceptions, and all made practical by a method logically de-

---

*Since this article has been put in type the newspapers have announced that the New York City Health Department has listed Tuberculosis as "an infectious and communicable disease," and has taken the first steps toward taking control of such cases and isolating them in great public institutions or hospitals. For many years the way has been gradually paved for taking this step, by which enormous power and patronage are gained.

duced from a study of the principles themselves, the whole confirmed by uniform success in practice for nearly a century.

Man is considered in his intellectual and spiritual as well as physical aspects. Life, which permeates his physical organism, conditioning every function, is identified with the infinite and universal life of God and its relation to environment defined. Disease is shown to be primarily simply a loss of harmony in the action between the component parts or planes of man's being, a purely dynamic disturbance, which is to be overcome by dynamic means, applied in accordance with a clearly defined and universally applicable remedial or harmonizing principle.

The seeker after truth has but to open his eyes to see. Examples of spontaneous restoration to health by the natural-divine method are seen almost daily. Analogies are too numerous to mention. Let him read the introduction to *The Organon*, with his mind open to the real purpose and aim of that masterly production, and learn there of the multitude of facts and observations, culled from the literature of the ages, which inducted Hahnemann into the knowledge of the law of cure.

Too often has the controversial form in which that production is cast been allowed to obscure the real meaning and purpose of the material there used. Let him remember that Hahnemann had the whole world of medicine to fight single-handed. Little wonder that the smoke of battle sometimes obscures the field of truth. Let him read the history of Hahnemann's personal experiments and demonstrations, conducted in accordance with the strictest requirements of scientific research that no error might creep in. Let him read the records of Hahnemann's self-sacrificing fellow-workers in this righteous cause, as they labored to perfect "an art whose end is the saving of human life." *Let him read the provings themselves*, in which these noble men and women, for the love of suffering humanity, not only suffered, but laid bare before the world their inmost thoughts and feelings, scorning the contumely and ridicule they knew would be theirs for a time. Let him read all this and feel the glow of enthusiasm mount and spur him on to emulation.

The high potency is the logical and necessary sequence of the dynamic philosophy of life and disease upon which the science of Homœopathy is based.

Similarity, or the principle of Assimilation, conditions all action throughout the universe. To assimilate is to become like or similar. All growth, all nutrition, all development, all action depends upon this. Similars unite, attract, repel, act and react. *Similia Similibus Curantur.* The form of the remedy must correspond to the form of the disease process. As the cause of the disease and the disease process itself are both dynamic in character, it follows that the remedy must also be dynamic in character and form. The remedy must not only be able to excite a similar action in the healthy organism, but in order to cure it must be raised by potentiation from the material to the dynamic plane upon which the cause of the disease is acting.

It may be said that crude medicines act curatively also, but experience shows that they act much more slowly and with greater pain and difficulty. "Aggravations" are more severe. In this case the organism itself attempts to potentiate the drug and raise it to the proper degree of curative power. The diluting media are the fluids and tissues of the body. The superfluous and useless material must be eliminated by vital processes. It is obnoxious to the organism, and the process of elimination is a painful one. Vitality is thereby wasted, the patient weakened, and the disease prolonged by thus forcing the organism to do for itself what should be done by art. The high potency saves time, suffering, and strength, because it is already raised to the same plane of action as that of the disease.

Read the history of the development of the idea of potentiation, that capstone of the noble structure which Hahnemann was erecting, and see in all this the struggle of a noble soul, divinely inspired, up out of the mists of ignorance and materialism which surrounded him into the light of transcendent truth, and reading, be yourself inspired.

Patiently, carefully, testing every step, Hahnemann evolved his marvelous Art of Healing; an art the like of which the world had never seen before; an art based upon the eternal laws of motion by which the teeming universe is governed. It germinated from a seed-thought in the mind of the master, planted while he laboriously translated the records of the meager knowledge of the action of Cinchona from the English of Cullen into his own tongue. It sprang up and sturdily grew into a mighty tree. It is growing yet.

As a system of philosophy, as well as of medicine, Homœopathy stands a complete, consistent, artistic whole. Every part fits into and corresponds with every other part. Each is essential to the whole and may not be left out without loss and injury.

Here, then, is a sphere in which the mind of the healing artist may revel. Here is a method by which the orderly operations of nature may be systematically and delightfully observed and studied. Here is a framework upon and around which the results of a lifetime in gathered facts and experiences may be arranged and classified. Here is room for self-culture and development of all the faculties, altruistic as well as egoistic. The powers of observation, perception, and conception; of analysis, synthesis, and comparison; of imagination; of reason, judgment, and memory, as well as the benevolent affections, all come into play in every case treated. In the oft-repeated, conscientiously accurate use of these faculties they may be enlarged and developed until they harmoniously unite and eventuate in the great all-inclusive faculty of INTUITION, through which we enter into true Seership—the open vision of the Truth.

